# Introduction of Pediatric Robot-Assisted Pyeloplasty in A Low-Volume Centre

**DOI:** 10.3390/clinpract11010020

**Published:** 2021-03-01

**Authors:** Niklas Pakkasjärvi, Seppo Taskinen

**Affiliations:** Department of Pediatric Surgery, New Children’s Hospital, Helsinki University Hospital, Stenbäckinkatu 9, 00029 HUS Helsinki, Finland; seppo.taskinen@hus.fi

**Keywords:** robot-assisted, pediatric pyeloplasty, cost analysis, minimally invasive, surgical training

## Abstract

(1) Background: This study investigated the introduction of pediatric robot-assisted pyeloplasty in a low-volume centre with reference to open pyeloplasty with regards to operative times, length of stay (LOS) and outcomes and cost analysis. (2) Methods: Data from 10 consecutive robot-assisted pyeloplasties was compared retrospectively to an age and weight matched cohort of open pyeloplasties operated on during two previous years. Operative times were analyzed in conjunction with LOS, outcomes and cost-analysis from patient records. (3) Results: Operative times remain longer in robot-assisted pyeloplasties (168 (IQR 68) vs. 141 (IQR 51) min), but patients are discharged from the hospital earlier and may return to daily activities earlier. In our hospital, the difference in LOS levels to some degree the cost difference between operations. (4) Conclusions: Robot-assisted pyeloplasty can be safely and economically introduced and maintained in a low-volume centre.

## 1. Introduction

While dismembered pyeloplasty has long been the standard of operative care for ureteropelvic junction obstruction, the advent of minimally invasive techniques is gaining merit. Smaller incisions and a shorter length of hospital stay (LOS) decrease the burden on the families involved. Minimally invasive approaches include laparoscopic pyeloplasty and robotic pyeloplasty. The learning curves of each are initially steep, but the long term outcomes seem equal to open procedures [1,2,3]. Although robot-assisted surgery offers many benefits over laparoscopy with increased dexterity of surgical instruments, improved 3D-viewing and optical magnification, one of the main drawbacks are the high costs associated with it. We sought to analyze the introduction of pediatric robot-assisted pyeloplasty in a low-volume centre (around 10 cases/year), to assess the viability of a new technique in a teaching hospital without prior experience in laparoscopic pyeloplasty. We also wanted to clarify the economic justification in low volumes of such an expensive approach.

## 2. Materials and Methods

### 2.1. Training

The robotic surgery program was instigated in pilot form in June 2019 and data was collected prospectively until November 2020 to evaluate the viability of such a program in a low volume center. The pediatric urology fellow had no prior laparoscopic pyeloplasty experience, but was very familiar with open renal surgery. The robotic console training consisted of 20 h of skill acquisition, followed by standardized training on pigs according to Intuitive Surgical protocols (Intuitive Surgical Inc., Sunnyvale, CA, USA). The fellow also visited the adult urologic clinic to follow robotic pyeloplasties and a designated adult surgeon was proctoring the first pediatric robotic pyeloplasty at our institute.

### 2.2. Patients

Indications for pyeloplasty were ureteropelvic junction obstruction detected by progressive hydronephrosis, worsening renal function or pain. During June 2019–May 2020 all patients over 12 kg or 2 years of age with indications for pyeloplasty were allocated a robotic-assisted operation. Eight patients presented with intermittent flank pain, 1 had progressive hydronephrosis and 1 had progressive hydronephrosis with infections. During this period, only one patient underwent open pyeloplasty. The operative registry at the division of pediatric surgery at the Helsinki University Central Hospital, University of Helsinki was reviewed for our first 10 consecutive patients operated for ureteropelvic junction obstruction with robot-assisted mini-invasive pyeloplasty during 2019–2020. The operative registry was then reviewed for patients who had undergone open pyeloplasty for ureteropelvic junction obstruction during 2017–2018, from which a cohort was age-matched to define a control group to the robot-assisted cohort. The patients were not randomized due to the retrospective nature of the study.

The operative registry was analyzed systematically with regards to sex, age, weight, preoperative urological measurements (AP-diameter, MAG3-renography), theatre time, operative time, console time, peri- and postoperative complications, postoperative urological measurements (AP-diameter, MAG3-renography) and reoperations. Complications were graded according to the Clavien–Dindo classification system [4].

Follow-up times were from 12 to 18 months postoperatively. Follow-up adherence was 100% for all patients.

### 2.3. Surgical Technique Robotic Pyeloplasty

All procedures were performed under general anesthesia with intravenous antimicrobial prophylaxis (cefuroxime 50 mg/kg). Pneumoperitoneum was achieved through an open technique infraumbilically. Robotic ports and docking were performed by manufacturer guided protocols. Three robotic ports were utilized (a 12 mm infraumbilical port and two 8 mm working ports) and one assisting 5 mm port. All robotic approaches were transabdominal. The DaVinci Si robotic surgical platform (Intuitive Surgical Inc., Sunnyvale, CA, USA.) was utilized for the robotics.

In right sided pyeloplasty, the kidney was approached by detaching the ascending colon. In left-sided pyeloplasty, a transmesenterial approach was utilized with direct access to the pelviureteric junction without any further colon mobilization. Stay sutures of 4-0 monofilament thread were placed in the pelvis and traditional Anderson–Hynes dismembered pyeloplasty was performed, with pelviureteral anastomosis and pyeloplasty done using 5-0 absorbable monofilament threads. Contrary to the traditional approach, the pelvic resection was smaller in the robotic group. A double-J stent of appropriate caliber and length was applied during surgery to be removed six weeks postoperatively. The transmesenterial opening was closed using the 4-0 monofilament thread and a continuous suture. Drains were placed to patients with mobilization of the colon and were removed at 24 h postoperatively. All patients had indwelling catheters for 24 h and were mobilized at own will. Prophylactic peroral antimicrobial treatment was continued until stent removal at one month postoperatively.

### 2.4. Surgical Technique Open Pyeloplasty

All open pyeloplasties were performed through an anterior extraperitoneal approach with standard Anderson–Hynes dismembered technique. Transcutaneous blue-stents were applied, which were subsequently removed at 1 week postoperatively.

### 2.5. Follow-Up

All patients attended postoperative follow-ups. JJ-stents applied during robotic surgery were removed six weeks postoperatively. A first follow-up appointment took place 3 months postoperatively with ultrasonography and MAG-3 renography. Clinical procedure included follow-ups with ultrasonography at 6 and 12 months postoperatively with additional follow-ups based on findings. The patients in the open group had attended follow-ups at 3 and 12 months with additional follow-ups based on findings.

### 2.6. Statistics

Continuous variables are described by median and interquartile ranges (IQR). Categorical variables are presented as frequencies and proportions. A Wilcoxon test or Mann–Whitney test was used for differences in medians, chi-squared or Fischer’s exact test was used for categorical variables. *p*-values of less than 0.05 were considered statistically significant.

### 2.7. Ethics and Consent

This study was approved by the Institutional Review Board at the New Children’s Hospital, Helsinki University Hospital, Helsinki, Finland. All work has been done in accordance with the Helsinki Declaration.

## 3. Results

Ten patients underwent robotic pyeloplasty during a nine month period 2019–2020, all of who were found to have extrinsic obstruction of the ureteropelvic junction. There were no conversions to open surgery, nor were there any perioperative complications. These patients were compared to an age- and weight-matched cohort of patients, who had undergone open pyeloplasty during two previous years at our institute (8.0 vs. 7.9 years and 28.3 vs. 29.5 kg; robotic vs. open cohort, respectively). The median operating time (from incision to skin closure) in the robotic group was 165 min (IQR 152–222), which was slower than the open pyeloplasty group (141 min (IQR 104–158), *p <* 0.05). The median console time was 124 min in the robotic group (IQR 115–170). The median length of hospital stay was 1.5 days (IQR 1–2) in the robotic pyeloplasty group and 6 days (IQR 4–7) in the open group (*p <* 0.05), see Table 1. None of the patients who underwent robotic pyeloplasty received epidural anesthesia postoperatively, which was in stark contrast to the open group, in which all but two had postoperative epidural anesthesia. The total amount of oxycodone administered to the patients during the hospital stay did not differ between groups (*p >* 0.05).

In both groups, alleviation of symptoms could be observed in all patients. Improvement in the degree of hydronephrosis could be detected in ultrasonography in all patients in the open cohort and in 9/10 in the robotic cohort at a 12 month follow-up. The rate of resolution was slower in the robotic group, with an average of 8 months to resolution compared to 5 months in the open group, but was not statistically significant (*p >* 0.05). MAG3-renographies showed resolution of obstruction in all but one in the robotic group, which was the same patient, who did not show improvement in hydronephrosis in ultrasonography. For this patient, a retrograde pyelography was performed, in which no clear obstruction could be observed. A filled 3F Fogarty embolectomy balloon with a diameter of 5 mm could easily pass over the ureteropelvic junction, suggesting that at least the anastomosis is patent (Edwards Lifesciences, Nyon, Switzerland). This patient had diminished function (13%) in the affected kidney preoperatively and had presented with infections. All patients reported alleviation of flank pain when that had been the indication for surgery (8/10 patients in the robotic cohort).

Three patients reported of discomfort due to stent related issues, either during micturition or during sports, which were resolved after stent removal and were deemed Grade 1 complications according to Clavien–Dindo. None of the patients in either group reported of any incision related issues. No reoperations were indicated during the study period.

To delineate the financial justification of robotic pyeloplasty in a low volume center, we investigated the costs related to surgery and hospital stay in our institute. The costs in our hospital for the surgical procedure itself are not dependent on the length of the procedure, but the costs for anesthesia and operating times are time dependent variables. For 2019, the cost of the robotic pyeloplasty including operating theatre, instruments and material was 6398 euros and that of open pyeloplasty 6076 euros. The total cost for robotic surgery for the operation and subsequent ward convalescence was 9213 euros, and for a traditional open pyeloplasty 8962 euros, converted to 2019 hospital rates. The difference in costs did not reach statistical significance (*p >* 0.1). The prices were revised yearly. The length of home convalescence was an approximation, but roughly the difference in groups was 4 days (verbal information and parents). Thus, given the current length of operating times and LOS including home convalescence, pediatric robotic pyeloplasty was efficient and economically justifiable at our institute. In the first patients, traditional double-J stents were used, which require an additional procedure for removal, which accounted for 2573 euros in total. However, we subsequently began to use magnetic stents, which avoided the need for anesthetic procedures, thereby limiting costs to only the material price for the stent as removal was scheduled simultaneously with the outpatient clinic.

## 4. Discussion

We show here, that the introduction of robot-assisted pyeloplasty to a low-volume centre was both feasible and economically justifiable. Both short- and mid-term outcomes were comparable to open surgery, but robotic pyeloplasty eases the burden on the families by a shorter hospital stay and smaller incisions and lower postoperative pain medication demands.

The robot-assisted pyeloplasty has gained a foothold within pediatric pyeloplasties especially within the USA. Operative times and outcomes seem comparable to open surgery, but economical aspects of the expensive robotic system has raised concerns. Seideman estimated that within the USA, robot-assisted pyeloplasty must be performed around 120 min and length of hospital stay limited to two days for economic justification [1]. Traditional laparoscopic pyeloplasty does not entail such strict demands due to cheaper instrumentation, but the learning curve of efficient laparoscopic knot-tying and suturing is steep. The learning curve for robot-assisted pediatric pyeloplasty has been estimated to be around 37 cases to achieve established operative times [5]. Sorensen et al. concluded that the operative time for robotic pyeloplasty time was within 1 SD of average open pyeloplasty after 15–20 cases [6]. Kassite further dissected particularities of the learning curve, and show that clear transition phases can be detected [7]. Although mastery regarding operative times may be attained after 16 cases, achieving mastery of a pediatric reconstructive procedure was estimated to require over 41 cases. In a Danish study, Reinhardt showed how robotic surgery could be initiated in low volume centers with a fellow as the console surgeon [8]. In line with our experience, they showed that start-up of a robotic program is possible with low volumes. Our volumes are even smaller, but still large enough to initiate robotic surgery in the light of current evidence. We opted to operate all pyeloplasties robotically if patients were over 2 years of age or over 12 kg in weight to be able to reach 10 cases within a reasonable time frame.

Salo and colleagues showed that robotic-assisted pediatric pyeloplasty is safe, with a similar success rate to open surgery, supporting robotic-assisted pyeloplasty in children [9]. They report a 100% success rate for hydronephrosis and flank pain. Their material is similar to ours, with roughly the same age and weight patients within the robotic group, but slightly longer operative times and length of hospital stay.

In our material, console times were two tailed, with issues with threads and stents occurring in three patients, while the operative times were uniform in the rest of the cases. While operative times were longer in the robotic group, the length of hospital stay was significantly shorter. The first patient in the robotic group opted to stay in the hospital for three days because of a long home journey (300 km), but patients over 20 kg tended to be discharged on the first postoperative day without any further encouragement within the robotic cohort. The amount of postoperative analgesia was lower in the robotic group, as none of the patients in this cohort received postoperative epidural analgesia, although the amount of per-oral oxycodone between groups was similar. The robotic cohort patients were allowed to return to daycare and school of their own free will without any demand for longer home convalescence. The open cohort patients were instructed to have a home convalescence of approximately one week after discharge, depending on circumstances. Taking the operative times, the length of stay at hospital and at home after discharge into consideration, the economic justification for robotic assisted surgery seems valid. Furthermore, maintaining robotic pyeloplasty practices with small volumes once initiated and proven viable seems equally acceptable. Low volumes regarding for instance nephrectomy are not linked to readmission rates and complication rates [10].

We operated two patients, in whom preoperative differential renal function (DRF) was <15% and thus had a poorly functioning kidney. After informed consent, the families opted for pyeloplasty in an attempt to at least postpone a possible nephrectomy. Postoperative renography did not reveal any significant improvement in DRF, but alleviation of symptoms could be observed by parents. One of these patients had preoperative infections and the postoperative renography did not show any improvement in DRF, in line with findings from Dowling and colleagues [11]. Gnech and colleagues report that pyeloplasty in poorly functioning kidneys rarely leads to nephrectomy during their mean follow-up of 91 ± 74 months [12]. Previous reports also support this finding, although in many cases, a preoperative percutaneous nephrostomy was utilized [13,14,15,16,17,18].

The introduction of robotic-assisted surgery demands appropriate dry training with dedicated consoles for technical skill acquisition [19]. We invested time on repetitive console training, both software based and surgical model training before advancing to proper surgery. The primary robotic surgeon also had experience of open surgery, with the assistant surgeon being extremely experienced. Prior to surgery, all steps were discussed with the team and constant evaluation of each step and stage during surgery was vocalized openly. In addition, procedures were analyzed in detail retrospectively and dry training was utilized to maintain a visual-proprioceptive memory of technique in-between robotic surgery.

Our study was not without problems. The number of cases was small and the two groups differed with regard to the experience level of the surgeon. The process for open surgery with everything involved has been leaned through many years of experience, while robotic surgery was completely new to us, encompassing many unforeseen retardations. We were unable to get dependable data on the home-convalescence as the study was done retrospectively, and the parents were asked for this data retrospectively in conjunction with the outpatient clinic. The follow-up is relatively short. Värelä and colleagues show, however, that follow-up beyond 12 months is not necessarily mandatory [20]. Despite these drawbacks, we believe that this pilot study still proves the viability of robotic surgery in our centre. We were able to utilize the robot from adult surgery without the need for initial acquisition, which of course have to be considered in the cost analysis in different settings. In a low volume-centre like ours, investing in a robot initially would naturally have been questionable. The operative process improved during progression, with subsequent reduction in costs. The average total costs for three patients, where everything proceeded according to plan was 7068 euros, indicating that through experience, benefits will likely materialize. There was no statistical difference in console times for robotic pyeloplasty vs. operation times for open pyeloplasty in our material. This has to be taken into consideration as epidural anesthesia was administered after the operation for the open pyeloplasty group, which was not taken into account in the operation times in contrast to the robotic group, where robotic docking was included in the operation times. Further, open surgery was retroperitoneal, while robotic surgery was transperitoneal or transmesenterial, which further introduces confounding factors. The transmesenterial approach appears to lower the risk for bowel disturbances and facilitate early discharge and fast recovery [21,22].

Our pilot study indicates that robotic pyeloplasty could safely be introduced into a low-volume center with limited laparoscopic renal surgery without significant financial burden (disregarding robot acquisition) or a need for long learning curves. The outcomes were similar to previous results and the burden to families was lower. With advancing experience, faster operative times are to be expected, further justifying the robotic approach for suitable patients. Training needs to be systematic and comprehensive for immediate introduction of the robotic system in a low volume center. Advancing proficiency in robotic surgery will also open new indications for its utilization.

## Figures and Tables

**Table 1:** Comparison of preoperative data, perioperative data and outcomes.

	Robotic Pyeloplasty	Open Pyeloplasty	*p*-Value
	*n* = 10	*n* = 10	
**Preoperative characteristics**			
Patient age	7.9	8.0	*p >* 0.05
Patient weight	28.3	29.5	*p >* 0.05
AP-diameter	28 (IQR 22–36)	21 (IQR 15–28)	*p >* 0.05
DRF	48 (IQR 30–50)	46 (IQR 34–49)	*p >* 0.05
**Perioperative data**			
Operative time minutes	165 (IQR 152–222)	141 (IQR 104–158)	*p <* 0.01
Console times	124 (IQR 115–170)	n/a	
Length of stay, days	1.5 (IQR 1–2)	6.0 (IQR 4–7)	*p <* 0.01
Opiates mg/kg (Oxycodone, po)	0.15 (IQR 0–0.4)	0.35 (IQR 0.07–0.7)	*p >* 0.05
Postoperative epidural anesthesia	0/10 patients	9/10 patients	*p <* 0.01
Total hospital charges of stay	9213 euros	8962 euros	
**Outcomes**			
AP-diameter reduction % at 1 year	67 (IQR 29–74)	36 (IQR 14–42)	*p >* 0.05
DRF	48 (IQR 30–51)	47 (IQR 40–50)	*p >* 0.05
Obstruction relieved in MAG3	9/10 patients	10/10 patients	*p >* 0.05
Symptom alleviation	10/10 patients	10/10 patients	*p >* 0.05

Data presented as medians and per patient unless otherwise stated. AP = Anteroposterior, DRF = differential renal function, IQR = interquartile range, PO = peroral

## Data Availability

Due to the nature of this research, data is not shared publicly, but can be discussed with corresponding author.
